# Description and Molecular Characterization of a New Dorylaimid Nematode, *Mesodorylaimus pini* n. sp. (Nematoda: Dorylaimidae) from Korea

**DOI:** 10.2478/jofnem-2024-0028

**Published:** 2024-09-30

**Authors:** Abraham Okki Mwamula, Sang Myeong Lee, Young Hak Jung, Yi Seul Kim, Dong Woon Lee

**Affiliations:** Research Institute of Invertebrate Vector, Kyungpook National University, Sangju 37224, Republic of Korea; SM Biovision Co., Jinju, 52849, Republic of Korea; Department of Entomology, Kyungpook National University, Sangju, 37224, Republic of Korea

**Keywords:** DNA barcodes, morphology, morphometrics, phylogeny, taxonomy

## Abstract

*Mesodorylaimus pini* n. sp., a new species isolated from the bark and cambium layer of a dead black pine tree is characterized herein using integrative taxonomy, considering both morphological and molecular phylogenetic analyses of the 18S- and 28S-rRNA genes. *Mesodorylaimus pini* n. sp. is characterized by having a medium-sized body 1.50–1.89 mm long; lip region angular and offset by a depression; a relatively long odontostyle (17.0–19.0 μm); vulval opening a transverse slit, positioned slightly posteriorly; *pars refringens vaginae* with two elongated drop-shaped to spindle-shaped sclerotizations; an intestine-prerectum junction with a long anteriorly directed conical or tongue-like projection; a relatively long female tail (115–187 μm); spicules 48.0–57.0 μm long; and regularly spaced 7–8 ventromedian supplements. It is closest to *M. subtilis*, especially in having similar body length and number of ventromedian supplements but can be differentiated from *M. subtilis* by the longer odontostyle, tongue-like projection, and longer spicules. The phylogenies based on the 18S- and 28S-rRNA sequences showed a well-supported sister relation of *M. pini* n. sp. with *M. subtilis, M. japonicus, M. bastiani, M. pseudobastiani, Calcaridorylaimus castaneae, C. heynsi*, and other member species of the group.

The genus *Mesodorylaimus* is one of the genera that were proposed by Andrássy (1959) to accommodate a considerable number of species whose morphological characters deviated from the type of *Dorylaimus* Dujardin, 1845, a genus that comprised a large and heterogeneous collection of species. *Mesodorylaimus* Andrássy, 1959 was erected to represent species with the following characteristics: body length usually between 1 and 2 mm; cuticle without protruding longitudinal ridges; lips relatively poorly developed, lip region slightly set off; moderately developed odontostyle; simple guiding ring; paired female gonads; vulva transverse and heavily sclerotized; the prerectum of the male significantly short, beginning in the area of the preanal supplements, rarely in front of the supplements; female tail elongated, pointed; and male tail short and bluntly rounded. In total, 39 species of *Dorylaimus* were transferred to *Mesodorylaimus*.

Since then, the genus has been amended several times by various taxonomists, and many species have either been added to the genus, making it one of the largest genera in the family Dorylaimidae or transferred to other new genera. A major revision of the genus was done by Andrássy (1986), including the erection of two new closely related genera (*Miodorylaimus* and *Calcaridorylaimus*) and providing keys for the identification of the valid species under *Mesodorylaimus* and these closely related genera. Andrássy (1988) further added seven more species and revised the key, providing a total list of 103 valid species. A number of new species have been described over the past years and the genus is currently represented by over 150 nominal species worldwide as detailed by Tauheed and Ahmad (2015), including the recently described *M. rivalis* and *M. ushkaniensis* (Gusakov and Gagarin, 2016; Naumova and Gagarin, 2021). The genus is cosmopolitan in distribution and comprises mainly terrestrial and freshwater species, and a few marine species. In Korea, two species, *M. usitatus* (Basson & Heyns, 1974) and *M. spengelii* (de Man, 1912; Andrássy, 1959) have so far been recorded (Geraert, 1997; Kang et al., 2021). During an ecological survey in the pine forest ecosystem in Korea, an undescribed species belonging to *Mesodorylaimus* was recovered from the bark and cambium layer of dead black pine (*Pinus thunbergii* Parl.) tree stands. The new species, *Mesodorylaimus pini* n. sp., is herein described using integrative taxonomy, considering both morphological and molecular phylogenetic comparisons.

## Materials and Methods

### Nematode population and extraction

The studied nematode population was extracted from the bark and cambium layer of a wilted and dead pinewood nematode-infected black pine (*Pinus thunbergii*) tree stand on Gonri Island in Tongyeong, Gyeongsangnam-do Province, Korea. Nematodes were extracted from the cuttings using the Baermann funnel method (Baermann, 1917). The collected nematode suspension was examined under a Nikon SMZ 1000 stereomicroscope (Nikon) and specimens belonging to *Mesodorylaimus* were picked out and subsequently characterized based on inferences from morphometric and DNA barcode data.

### Morphological characterization

The 31 female and 32 male specimens of the recovered population were heat-killed, fixed, and mounted to pure glycerin (Seinhorst, 1959). Morphometric data and photomicrographs were taken using a Zeiss Imager Z2 microscope (Carl Zeiss) fitted with AxioVision, a material science software for research and engineering (Carl Zeiss). Line drawing of the specimens was made under a drawing tube before being redrawn using CorelDRAW® software version 24. Species delineation and diagnosis were done following the keys presented by Andrássy (1986, 1988) and the various original species descriptions by Loof (1969); Thorne (1974); Botha and Heyns (1992); Ahmad (1993); Nedelchev and Peneva (2000); Peña Santiago and Abolafia (2000); and Tauheed and Ahmad (2010).

### Molecular characterization

Ribosomal DNA was extracted from single female, male, and juvenile specimens using the DNA extraction kit WizPure™, as described by Iwahori et al. (2000). The nearly full-length 18S-rRNA gene was amplified as two partially overlapping fragments using two primer sets: 988F (5′-CTCAAAGATTAAGCCATGC-3′) and 1912R (5′-TTTACGGTCAGAACTAGGG-3′), 1813F (5′-CTGCGTGAGAGGTGAAAT-3′) and 2646R (5′-GCTACCTTGTTACGACTTTT-3′), (Holterman et al., 2006). The D2A (5′-ACAAGTACCGTGAGGGAAAGTTG-3′) and D3B (5′-TCGGAAGGAACCAGCTACTA-3′) primer set (Nunn, 1992) was used in the amplification of the D2-D3 expansion segment of 28S-rRNA. Polymerase chain reaction (PCR) was performed with a PCR cycler (T100™, Bio-Rad), and the PCR thermal profiles for the D2A/D3B, 988F/1912R, and 1813F/2646R primer sets were as described by Mwamula et al. (2023a). The PCR products were purified using a PCR purification kit (Qiagen) and quantified using a quickdrop spectrophotometer (Molecular Devices). The purified PCR products were subsequently used for direct sequencing in both directions using the same primers as specified above. DNA sequencing was done at Macrogen. The edited DNA sequences were submitted to the GenBank database under the accession numbers PP525782-PP525784 (for 18S-rRNA) and PP525785-PP525788 (for 28S-rRNA).

### Phylogenetic analysis

The edited sequences (18S-rRNA and 28S-rRNA gene) were aligned using ClustalX (Thompson et al., 1997) along with the sequence data sets of *Mesodorylaimus, Calcaridorylaimus*, and other species from related genera that are published in GenBank (Holterman et al., 2006; Nedelchev et al., 2014; Vinciguerra et al., 2016; Kagoshima et al., 2019; Swart et al., 2020; Zhang et al., 2023). The partial sequences of *Paravulvus hartingii* (de Man, 1880; Heyns, 1968) (AY552976) and *Nygolaimus cf. parvus* (Thorne, 1974) (AY552974) were selected as the outgroup taxa for the 18S-rRNA gene. *Mononchus tunbridgensis* (Bastian, 1865) (AY593063) and *Anatonchus tridentatus* (de Man, 1876; De Coninck, 1939) (AY593065) were the outgroup for 28S-rRNA gene. The generated alignments were analyzed with Bayesian Inference (BI) using MrBayes 3.2.7 (Ronquist et al., 2012) under the GTR + I + G model. Bayesian inference analysis for each gene was initiated with a random starting tree and run with four chains for 1 × 10^6^ generations. The Markov chains were sampled at intervals of 100 generations. After discarding burn-in samples, a consensus tree was generated with a 50% majority rule. The generated trees were visualized and edited using FigTree v1.4.4 software. Posterior probabilities (PP) exceeding 50% are given on appropriate clades. Intraspecific and interspecific sequence variations were examined using PAUP* v4.0a169 (Swofford, 2003).

## Results

**Figure 1.**
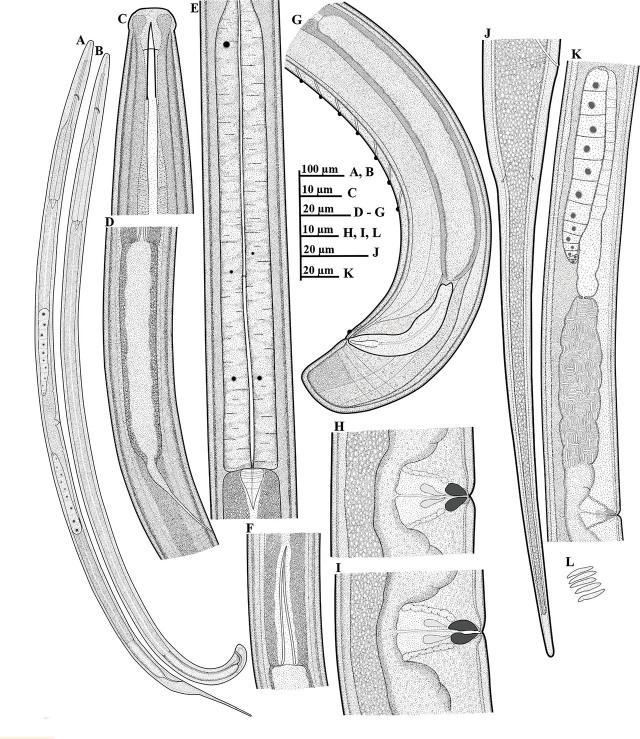
Illustrations of *Mesodorylaimus pini* n. sp. (A-L): A: Female whole body; B: Male whole body; C: Female anterior region; D: Female rectum and prerectum region; E: The expanded part of pharynx; F: Junction between intestine and prerectum with a long conical or tongue-like structure; G: Posterior end of male, including copulatory apparatus and the arrangement of ventromedian supplements; H, I: Vulval region, with variation in shape of sclerotizations; J: Female tail region; K: Female reproductive system; L: Sperm cells in testis and uterus.

**Figure 2.**
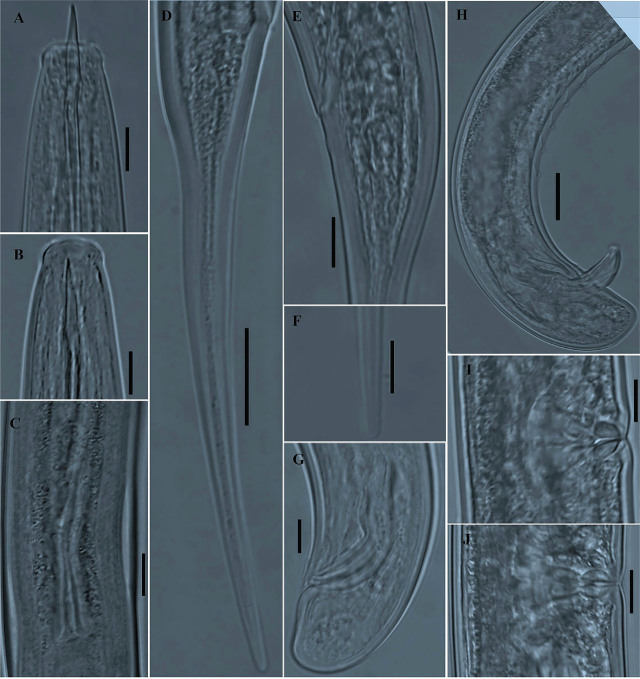
Photomicrographs of *Mesodorylaimus pini* n. sp. (A-J). A, B: Variation in female anterior region; C: Junction between intestine and prerectum with a long conical or tongue-like structure; D, E, F: Variation in female tail region; G: Male tail region; H: Male posterior region with ventromedian supplements and copulatory apparatus; I, J: Vulval region and variation in shape of sclerotizations (Scale bars: A, B, C, E, F, G, I and J = 10 μm; D and H = 20 μm).

*Mesodorylaimus pini* n. sp. (Figs. 1 & 2)

### Measurements:

See Table 1.

**Table 1.** Morphometrics of *Mesodorylaimus pini* n. sp. from Korea

**Characteristic**	**Holotype** ♀	♀♀	♂♂

**n**		**31**	**32**
L	1802	1702.6 ±101.5 (1503–1889)	1558.2 ± 103.0 (1366–1775)
a	44.5	43.0 ± 2.2 (37.7–47.0)	40.6 ± 2.2 (35.3–44.9)
b	4.8	4.6 ± 0.3 (4.0–5.1)	4.3 ± 0.2 (3.6–4.8)
c	10.6	11.9 ± 1.0 (10.0–13.6)	73.1 ± 8.1 (56.3–87.5)
c’	6.5	5.8 ± 0.6 (4.9–7.0)	0.8 ± 0.1 (0.6–0.8)
V	50.5	51.4 ± 1.2 (50.0–54.9)	_
G1%	17.4	17.8 ± 1.7 (14.3–19.6)	_
G2%	17.8	18.6 ± 1.8 (14.6–20.7)	_
Lip height	5.0	5.0 ± 0.4 (4.5–6.0)	5.2 ± 0.5 (4.5–6.0)
Lip diam.	12.5	12.2 ± 0.5 (11.0–13.5)	12.3 ± 0.5 (11.0–13.0)
Anterior to guiding ring	9.0	9.2 ± 0.3 (8.5–9.5)	9.2 ± 0.2 (9.0–9.5)
Odontostyle	18.0	18.2 ± 1.0 (17.0–19.0)	18.1 ± 0.9 (17.0–19.0)
Odontophore	21.5	22.0 ± 1.2 (19.0–24.0)	21.7 ± 1.5 (19.0–23.5)
Total spear	39.5	40.2 ±1.7 (36.0–42.5)	39.9 ±1.9 (36.0–42.0)
Anterior to nerve ring	127.0	126.5 ± 5.2 (119.0–143.0)	124.4 ± 2.7 (118.0–132.0)
Pharynx length	377	369.2 ± 16.7 (333–398)	358.7 ± 16.7 (326–390)
Expanded part of pharynx	160	161.3 ± 9.6 (142.0–186.0)	151.2 ± 11.1 (133.0–179.5)
Cardia length	21.0	20.1 ± 3.6 (14.5–27.5)	20.8 ± 3.3 (14.0–25.5)
Maximum body diam.	40.5	39.7 ± 2.2 (32.0–43.5)	38.4 ± 2.1 (33.0–42.0)
Vulva to anus	739	729.2 ± 35.3 (647–817)	_
Prerectum	78.5	80.8 ± 6.5 (69.0–99.0)	119.2 ± 9.1 (110.5–151.5)
Rectum	31.5	32.0 ± 2.6 (28.0–38.0)	44.0 ± 4.0 (35.0–49.5)
Anal / cloacal body diam.	26.0	24.7±1.3 (22.0–26.5)	28.5 ± 1.6 (25.0–32.0)
Tail length	179.0	144.4 ± 18.4 (115.0–187.0)	21.5 ± 1.8 (18.0–25.0)
Spicules	_	_	52.3 ± 1.8 (48.0–57.0)
No. of ventromedian supplements	_	_	7–8

### Description

*Female (n = 31):*

General habitus is slightly curved ventrally. Cuticle is 2–3 μm thick at the base of pharynx, 3–4 μm at midbody, and 4.0–5.5 μm at postanal region. Lateral chords are *ca* 1/3–1/4 of body diameter. Lips are angular, with lip region offset by a depression, 11.0–13.5 μm wide, 4.5–6.0 μm high. The amphidial aperture is wide, 5.5–6.5 μm wide or about half as wide as the lip region diameter; the amphidial fovea is typically stirrup-shaped. Odontostyle is typical of the genus, *ca* 1.4–1.7 times the lip region diameter, and its aperture occupies 28%–37% of its length. Odontophore are simple, rod-like, *ca* 1.1–1.3 times the odontostyle length. The guiding ring is thin and single, located at 8.5–9.5 μm from the anterior end. The nerve ring encircles the corpus part of the pharynx at *ca* 31–38% of total pharynx length from anterior end. The pharynx is 333–398 μm long, *ca* 0.5–0.6 times as long as the distance between pharynx and vulva, posteriorly expanding to a muscular expanded part. Pharyngeal expansion begins at ca 52–59% of the total neck length, appearing almost uniform in width along its length and slightly wider at the posterior end. Body diameter at the posterior end of pharyngeal expansion is *ca* 3 (2.7–3.5) times the lip region diameter. DN large, 4.0–4.5 μm, globular, is located near the anterior end of the expanded part, 10–25 μm from the anterior-most part of the expanded part. The two S_1_N are located in the posterior half of the distance DN-S_2_N, the anterior one (S_1_N_1_) appearing smaller (1.5–2 μm in diameter) than S_1_N_2_ (2–4 μm in diameter). S_2_N visible, globular shaped, 2–3 μm *in* diameter located in the posterior portion of the expanded part. The pharyngeal gland nuclei with their orifices are located as follows: (n = 8): DO = 57–59; DN = 60–62; DO-DN = 2.1–2.8; S_1_N_1_ = 79–82; S_1_N_2_ = 81–84; S2N = 88–91; S2O = 90–92 (for terminology and formulae, see Loof and Coomans (1970)).

Cardia is typically conoid, variable in length, 14.5–27.5 μm long. Reproductive system is didelphic-amphidelphic. Ovaries are reflexed, generally variable in length, not reaching the vulva level, and identical as illustrated in Figure 1A. The anterior branch occupies 14–20% of the total body length, and the posterior branch 15–21%. Each branch is basically arranged from ovary to gonoduct (oviduct outstretched, joining ovary sub-terminally; sphincter weak, present at oviduct-uterus junction, joining an undifferentiated uterus) and vagina. The uterus is filled with well-developed, elongated, and sausage-shaped sperms. The vulva is a transverse slit. *Pars distalis vaginae* 1.5–2.5 μm long. *Pars refringens vaginae* has two elongate drop-shaped to spindle-shaped sclerotizations, each measuring 5.0–8.0 μm deep and 2.5–3.5 μm wide, with a combined width of 6.0–9.5 μm. *Pars proximalis* are 6.0–9.0 μm wide and 12.5–16.0 μm long. Intestine-prerectum junction has a long anteriorly directed conical or tongue-like projection measuring 32–50 μm long, or about 1.1–1.7 times as long as the corresponding body diameter. The rectum and prerectum are *ca* 1.1–1.5 and 3.0–4.3-times anal body diameter long, respectively. Anterior tail part (*ca* 25–38 μm from anal opening) is convex-conoid, with two pairs of caudal pores, one subventral and the other subdorsal; the tail continues gradually and tapers to a smoothly rounded tail terminus. The posterior part of the tail appears straight. Total tail length *ca* 4.9–7.0 times anal body diameter.

*Male (n = 32):*

They are similar to females in general morphology except for sexual characteristics and short conoid tail. Their body is curved ventrally to open in a J-shape when heat-killed and fixed. The genital system is diorchic, and testes are opposed and well-developed with well-developed elongated and sausage-shaped sperms. Spicules are ventrally curved, 48–57 μm long, or *ca* 1.7–2.1 times the cloacal body diameter. Lateral guiding pieces are 10.0–13.5 μm long, tapering at the end. Supplements consist of an adcloacal pair and 7–8 regularly spaced ventromedians. The posterior-most ventromedian supplement located at 33–50 μm from the adcloacal pair. Intestine-prerectum junction begins mostly at the level of the last supplement or slightly extending beyond the range of supplements by 0–25 μm. The rectum and prerectum are *ca* 1.2–1.8 and 3.5–5.4-times cloacal body diameter long, respectively. The tail appears dorsally conoid and broadly rounded. Tail length is less than cloacal body diameter (*ca* 0.6–0.8 times cloacal diameter).

### Diagnosis and relationships

*Mesodorylaimus pini* n. sp. is characterized by having a medium-sized body (L = 1.50–1.89 mm), lip region angular and offset by a depression; a relatively long odontostyle (17.0–19.0 μm), 1.4–1.7 times as long as the lip region diameter; expanded part of pharynx 142–186 μm long, vulval opening a transverse slit, positioned slightly posteriorly (V = 50–55); *pars refringens vaginae* with two elongated drop-shaped to spindle-shaped sclerotizations; intestine-prerectum junction with a long anteriorly directed conical or tongue-like projection, 1.1–1.7 times as long as the corresponding body diameter; a relatively long female tail (115–187 μm, c = 10.0–13.6; c’ = 4.9–7.0), spicules 48.0–57.0 μm long, 2–3 times the tail length; and the regularly spaced 7–8 ventromedian supplements.

*Mesodorylaimus pini* n. sp. is very close to *M. subtilis* (Thorne & Swanger, 1936; Andrássy, 1959), *M. subtiliformis* (Andrássy, 1959; Andrássy 1959), *M. pseudosubtilis* (Basson & Heyns, 1974), *M. shamimi* (Ahmad & Araki, 2003), and *M. noreasus* (Tauheed & Ahmad, 2010). It differs from all by the presence of a long anteriorly directed conical or tongue-like projection in the intestine-prerectum junction. In addition, *Mesodorylaimus pini* n. sp. differs from *M. subtilis* by the relatively longer body length (1.37–1.78 *vs* 1.3 mm in males and 1.50–1.89 *vs* 1.4–1.5 mm in females), longer odontostyle (17.0–19.0 μm *vs* 12 μm), shorter tail (c = 10.0–13.6, c’ = 4.9–7.0 *vs* c = 8.3–8.5, c’ = 8), a relatively more posteriorly positioned vulva (V = 50–55 *vs* 44–50), tail tapering to a smoothly rounded tail terminus *vs* subfiliform, and longer spicules (*ca* 2–3.0 times tail length *vs* hardily longer than the tail); from *M. subtiliformis* by the longer body length (1.50–1.89 *vs* 1.3 mm), lip region set off by a depression *vs* not set off or only barely set off, longer odontostyle (17.0–19.0 μm *vs* 14–15 μm), a straight tail that tapers to a smoothly rounded tail terminus *vs* filiform and pointed at the terminal end, longer spicules (48–57 μm *vs* 44.5 μm), and relatively fewer number of ventromedian supplements (7–8 *vs* 10); from *M. pseudosubtilis* by longer odontostyle (17.0–19.0 μm *vs* 10–14 μm), a relatively more posteriorly positioned vulva (V = 50–55 *vs* 46–50), tail tapering to a smoothly rounded tail terminus *vs* filiform with acute terminus, longer spicules (48–57 μm *vs* 41 μm), a relatively fewer number of ventromedian supplements (7–8 *vs* 10), and the prerectum-intestine junction either slightly anterior to range or almost at level of ventromedian supplements *vs* prerectum terminating well within the range of supplements; from *M. shamimi* by the relatively longer body length (1.37–1.78 *vs* 1.12–1.29 mm in males and 1.50–1.89 *vs* 1.2–1.52 mm in females), longer odontostyle (17.0–19.0 μm *vs* 13.5–16 μm), *pars refringens vaginae* with two elongated drop-shaped to spindle-shaped sclerotizations, 5.0–8.0 μm deep *vs* short (4.0–4.5 μm) triangular sclerotization pieces, female tail tapering to a smoothly rounded tail terminus *vs* acute terminus, longer prerectum in males (111–152 μm *vs* 105 μm), and relatively longer spicules (48–57 μm *vs* 45–49 μm); and from *M. noreasus* by longer odontostyle (17.0–19.0 μm *vs* 14–16 μm), *pars refringens vaginae* with two elongated drop-shaped to spindle-shaped sclerotizations, *vs* two well separated rectangular sclerotizations, longer prerectum in females (3.0–4.3 times the anal body diameter *vs* 2.4–3.0 times the anal body diameter), longer spicules (48–57 μm *vs* 42–43 μm), and the prerectum-intestine junction either slightly anterior to range or almost at level of ventromedian supplements *vs* prerectum extending far beyond the range of supplements.

Among species with a long anteriorly directed conical or tongue-like projection in the intestine-prerectum junction, *Mesodorylaimus pini* n. sp. is close to *M. trapaefructus* Andrássy, 1986; *M. baeticus* Peña-Santiago & Abolafia, 2000; and *M. nevadensis* Peña-Santiago & Abolafia, 2000. *Mesodorylaimus pini* n. sp. differs from *M. trapaefructus* by the lip region shape (lip region angular and offset by a depression, wider than the adjacent neck region *vs* narrowing head, not offset, narrower than adjacent neck region), *pars refringens vaginae* with two elongated drop-shaped to spindle-shaped sclerotizations *vs* heavily cuticularized and peculiarly characteristic in shape, resembling the crop of the water-chestnut, longer tail, posterior part straight with a smoothly rounded terminus *vs* shorter tail, spicate posterior part always curved dorsally (c = 10.0–13.6 *vs* c = 14–18), longer prerectum (3.0–4.3 times anal body diameter *vs* 2.6–2.8 times as long as anal body diameter), thick cuticle 3–4 μm at mid-body, and 4.0–5.5 μm at postanal region *vs* cuticle smooth and thin, 1.5 μm on mid-body and 2.5 μm on anterior end of tail, and males abundant *vs* no males; from *M. baeticus* by lip region set off by a depression *vs* lip region continuous or offset by a weak depression, longer odontostyle (17.0–19.0 μm *vs* 13–15 μm), relatively longer tail, convex-conoid, continuing gradually, and tapering to a smoothly rounded tail terminus *vs* tail elongated, tapering abruptly at first, then more gradually to a cylindrical thickened terminal portion (c = 10.0–13.6 *vs* c = 12–19), and males abundant *vs* no males; and from *M. nevadensis* by the long odontostyle (17.0–19.0 μm *vs* 12.5–14 μm), cardia typically conoid variable in length *vs* cardia enveloped by intestinal tissue forming a tongue-like extension, long spicules (48–57 μm *vs* 33.5–37.5 μm in one population and 41–47 μm in paratypes), and the *pars refringens vaginae* with two elongated drop-shaped to spindle-shaped sclerotizations, 5.0–8.0 μm deep *vs pars refringens vaginae* with two divergent and usually well separated triangular to drop-shaped sclerotizations measuring 3 x 5 μm.

### Type of habitat and locality

The nematode population was recovered from the bark and cambium layer of a wilted and dead pinewood nematode-infected black pine (*Pinus thunbergii*) tree stand on Gonri Island in Tongyeong, Gyeongsangnam-do Province, Korea. GPS coordinates: 34°46′38″N, 128°21′45″E).

### Type of material

Holotype female, 16 female, and 18 male paratypes were deposited in the Nematode Collection of Kyungpook National University (KNU), South Korea; 10 female and 10 male paratypes were deposited in the Nematode Collection of Makerere University, Kampala, Uganda; and 5 females and 4 males were deposited in the Nematode Collection in the Ghent University Zoology Museum, Gent, Belgium.

### Etymology

*Mesodorylaimus pini* n. sp. was isolated from the bark and cambium layer of a dead black pine tree. Thus, the species epithet *pini* is derived from the tree name.

### Molecular characterization and phylogenetic relationships

The amplification of the nearly full-length 18S-rRNA and the D2-D3 expansion segment of the 28S-rRNA gene yielded single fragments of approximately 1,700 and 780 bp, respectively. The three newly obtained 18S-rRNA gene partial sequences (PP525782-PP525784) exhibited no intraspecific variation (0.0%), and the top 5 hits in GenBank BLAST homology search for these three sequences comprised closely homologous sequences of unidentified *Mesodorylaimus* isolate (MG921251), *Mesodorylaimus cf. nigritulus* (AJ966490), *Calcaridorylaimus* isolates (MG921242 and MG921244), *Ecumenicus* sp. (MK292127), and *Aporcelaimellus obtusicaudatus* (DQ141212), all with percent identities of over 98%. In the 18S-rRNA gene phylogeny, *Mesodorylaimus pini* n. sp. sequences were grouped in a well-supported subclade (PP = 100) with *M. pseudobastiani* (AY919195), *Mesodorylaimus cf. simplex* (AY146504)*, Calcaridorylaimus signatus* (LC457654), *Mesodorylaimus* sp. (MG921251), *Calcaridorylaimus* sp. (MG921242), *Calcaridorylaimus* sp. (MG921244), *Mesodorylaimus cf. nigritulus* (AJ966490), *Mesodorylaimus japonicus* (AJ966489), *M. bastiani* (AJ966488 and MF325117), *Mesodorylaimus subtilis* (MG921247), and *Calcaridorylaimus castaneae* (KF717497) differing by 8 bp (1.2%), 9 bp (1.4%), 13 bp (1.5%), 27 bp (1.6%), 27 bp (1.6%), 28 bp (1.6%), 29 bp (1.7%), 33 bp (1.9%), 21–39 bp (2.4–2.8%), 47–48 bp (2.8%), and 47 bp (2.9%), respectively.

**Figure 3.**
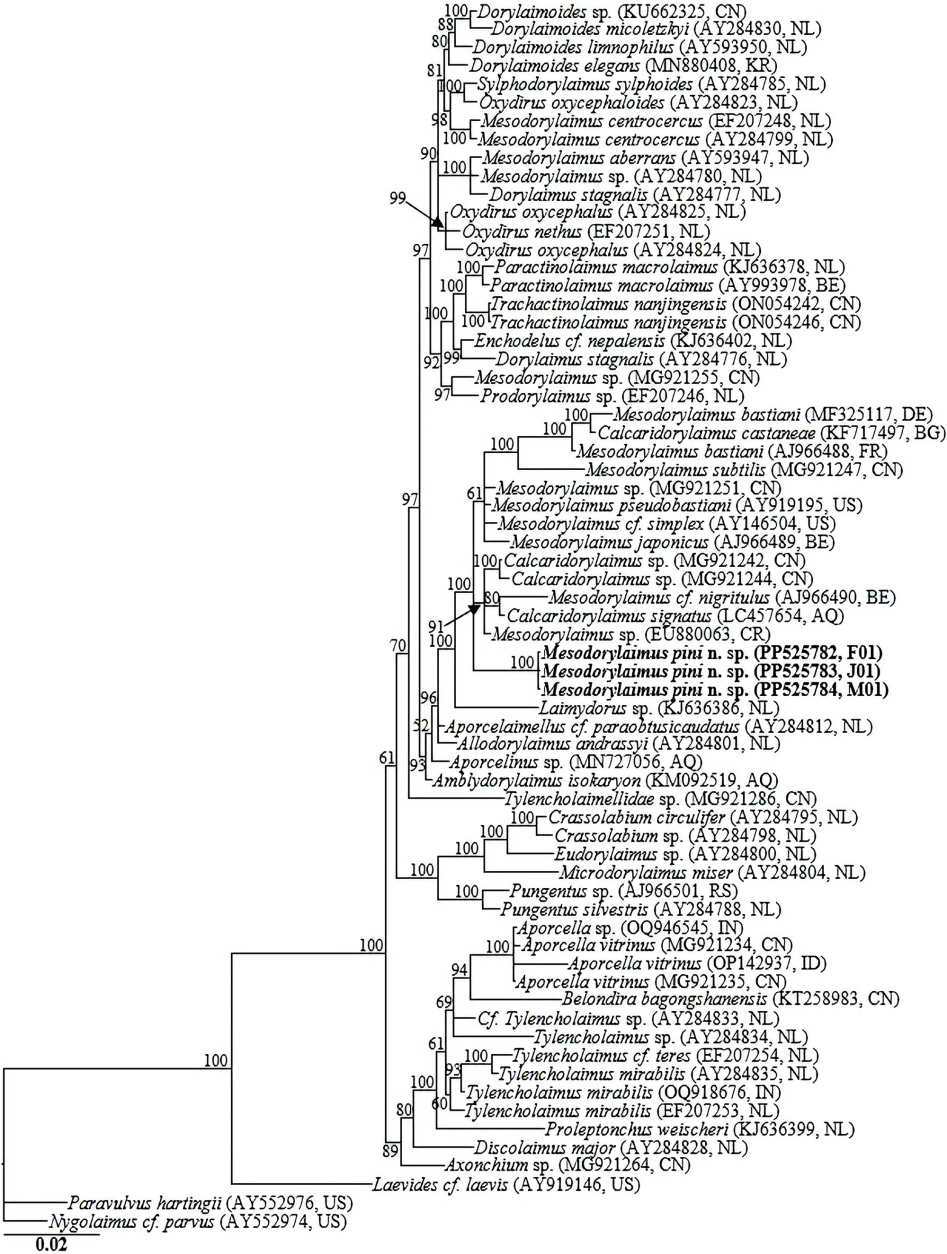
Bayesian tree inferred under the GTR + I + G model from 18S-rRNA gene sequences of Dorylaimid species. Posterior probability values exceeding 50% are given on appropriate clades. The studied population is indicated in bold text. Outgroup taxa: *Paravulvus hartingii* and *Nygolaimus cf. parvus*.

**Figure 4.**
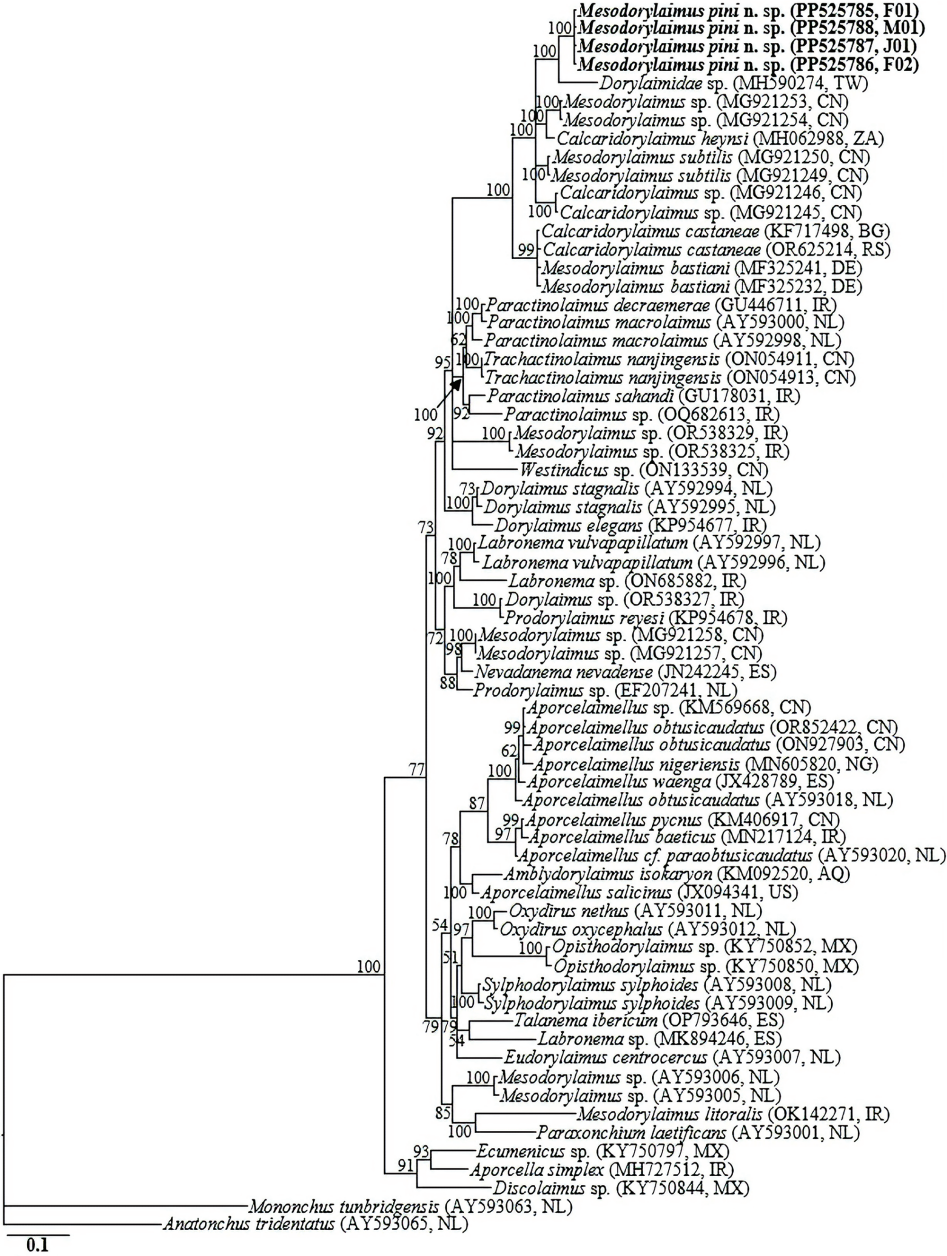
Bayesian tree inferred under the GTR + I + G model from LSU D2–D3 partial sequences of Dorylaimid species. Posterior probability values exceeding 50% are given on appropriate clades. The studied population is indicated in bold text. Outgroup taxa: *Mononchus tunbridgensis* and *Anatonchus tridentatus*.

The four D2-D3 sequences from the different life stages and sexes of *Mesodorylaimus pini* n. sp. (PP525785-PP525788) were identical, with no intraspecific sequence variation (0.0%). Similar to 18S-rRNA gene phylogeny, the D2-D3 sequences of *Mesodorylaimus pini* n. sp. were grouped in a well-supported subclade with the sequences from *Mesodorylaimus bastiani* isolates (MF325232 and MF325241), an unidentified *Mesodorylaimus* sp. (MG921253 and MG921254), *Mesodorylaimus subtilis* (MG921249 and MG921250), Dorylaimidae sp. (MH590274), *Calcaridorylaimus heynsi* (MH062988), *Calcaridorylaimus* sp. (MG921245 and MG921246), and *Calcaridorylaimus castaneae* (KF717498 and OR625214), differing by 17 bp (5.3%–5.5%; with 39.8% coverage), 43 bp (5.6%), 44 bp (5.7%), 44 bp (6.0%), 48 bp (6.6%), 50 bp (6.5%), and 71 bp (9.2–10.0%), respectively. Sixty-seven 18S-rRNA and 67 28S-rRNA gene sequences from various member species of *Mesodorylaimus, Calcaridorylaimus* and other related genera, inclusive of the newly obtained sequences and outgroup taxa, constituted the sequence dataset for phylogenetic analyses. Phylogenetic relationships, as inferred from Bayesian analysis of the dataset with GTR + I + G substitution model, are shown in Figures 3 and 4.

## Discussion

Analyses of 18S-rRNA and the D2-D3 expansion of 28S-rRNA gene strongly suggest that *Mesodorylaimus pini* n. sp. is distinct from the few available *Mesodorylaimus* gene sequences as represented by the Bayesian trees. The phylogenetic inferences suggest that it is a sister species to the morphologically close *M. subtilis* and other *Mesodorylaimus* spp, including *M. bastiani, M. pseudobastiani*, and *M. japonicus*. Also, in the two phylogenies, *Mesodorylaimus* species clustered with populations of *Calcaridorylaimus* spp. in well-supported subclades, including *C. signatus, C. castaneae*, and *C. heynsi*. This agrees with the close morphological similarities between the two genera. Morphologically, apart from the unique shape and structure of spicules (the double contour on the dorsal side and a spur before the tip of spicules) in *Calcaridorylaimus*, other diagnostic characteristics for the two genera, especially in females, are indistinguishable.

Integrative taxonomy considering both morphological characteristics and DNA barcodes provides a better-supported approach to nematode identification (Subbotin, 2015; Mwamula et al., 2023b). However, these non-parasitic nematodes have not been given the necessary attention, and DNA sequence data of many of these species are still unavailable. Similar to the few others available, and the related studies of Nedelchev et al. (2014) and Swart et al. (2020), the phylogenetic topologies in the current study showed the polyphyly nature of *Mesodorylaimus*. However, it should be noted that these conclusions are based on a very limited number of sequences available in the NCBI GenBank. Due to the insufficient number of available DNA barcodes for the group in all public databases, accurate phylogenetic inferences cannot be drawn with absolute certainty. In conclusion, molecular characterization of the various species of the two genera and other related groups is necessary to aid the reconstruction of phylogenetic patterns within the group. This will supplement the current generic compendia.
